# Improved Species-Specific Lysine Acetylation Site Prediction Based on a Large Variety of Features Set

**DOI:** 10.1371/journal.pone.0155370

**Published:** 2016-05-16

**Authors:** Qiqige Wuyun, Wei Zheng, Yanping Zhang, Jishou Ruan, Gang Hu

**Affiliations:** 1 School of Mathematical Sciences and LPMC, Nankai University, Tianjin, China, 300071; 2 Department of Mathematics, School of Science, Hebei University of Engineering, Handan, China, 056038; 3 State Key Laboratory of Medicinal Chemical Biology, Nankai University, Tianjin, China, 300071; Harbin Institute of Technology Shenzhen Graduate School, CHINA

## Abstract

Lysine acetylation is a major post-translational modification. It plays a vital role in numerous essential biological processes, such as gene expression and metabolism, and is related to some human diseases. To fully understand the regulatory mechanism of acetylation, identification of acetylation sites is first and most important. However, experimental identification of protein acetylation sites is often time consuming and expensive. Therefore, the alternative computational methods are necessary. Here, we developed a novel tool, KA-predictor, to predict species-specific lysine acetylation sites based on support vector machine (SVM) classifier. We incorporated different types of features and employed an efficient feature selection on each type to form the final optimal feature set for model learning. And our predictor was highly competitive for the majority of species when compared with other methods. Feature contribution analysis indicated that HSE features, which were firstly introduced for lysine acetylation prediction, significantly improved the predictive performance. Particularly, we constructed a high-accurate structure dataset of *H*.*sapiens* from PDB to analyze the structural properties around lysine acetylation sites. Our datasets and a user-friendly local tool of KA-predictor can be freely available at http://sourceforge.net/p/ka-predictor.

## Introduction

**Fig 1:**
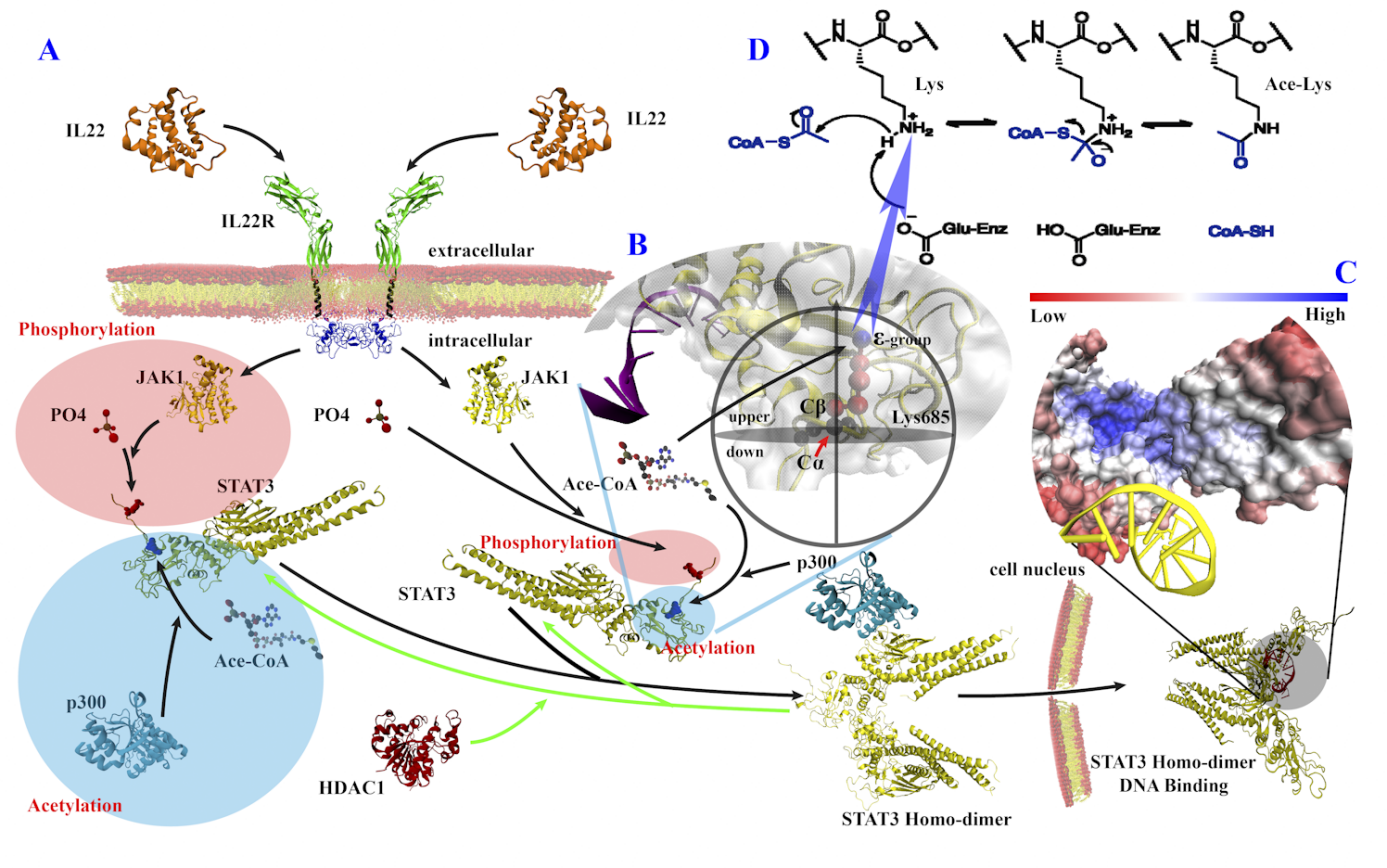
The importance of acetylation of STAT3 (pdb:1BG1) and lysine acetylation equation. (A) Jak-STAT signaling pathway, including phosphorylation and acetylation. (B) The schematic diagram of HSE. An acetyl group (CH_3_CO-) from Ace-CoA replaces the hydrogen atom in the epsilon-amino group (-NH_3_^+^)(blue) of side chain (red) of lysine residue, and the side chain of Lys685 is located at the upper half sphere which contains C_β_ atom. (C) DNA binds to STAT3 homo-dimer, and surface of STAT3 is colored by CHOPS[5] score, a convex measure of protein surface. (D) Lysine acetylation equation.

Acetylation is one of the most significant post-translational modifications of proteins, and plays an important role in various cellular processes [1–3], such as cytokine signaling, transcriptional regulation and apoptosis. Acetylation typically occurs on lysine residues describing a process of introducing an acetyl group (CH_3_CO-) into the side chain of an amino acid in a protein. This reaction is a reversible modification relying heavily on various enzymes. Lysine acetylation is catalyzed by histone acetyltransferases (HATs) or lysine acetyltransferases (KATs), which transfer the acetyl-group to the epsilon-amino group of a lysine residue (**Fig 1D**), while lysine deacetylation by histone deacetylases (HDACs) or lysine deacetylases (KDACs) that remove the acetyl-groups[4].

Acetylation has a considerable impact on gene expression and metabolism[6], and is also related to some human diseases[7,8]. For example, signal transducer and activator of transcription 3 (STAT3) is a transcription factor. The lysine acetylation of STAT3 is of key importance in the DNA-binding and the transcription for the oncogenic activity which is related to many cancers such as pancreatic cancer[9] (**Fig 1**). In addition, the level of acetylated STAT3 is relatively high in cancer cells[10]. Therefore, it may be a potential and promising strategy to target acetylated STAT3 in cancer therapy.

Since the acetylation plays an important role in the cell biology and diseases treatment, it is really essential to understand the regulatory mechanism of acetylation. Thus, the first step is to identify the acetylation sites. Various experimental methods have been developed to identify the potential lysine acetylation sites of a protein, such as radioactivity detection[11], mass spectrometry[12], and chromatin immunoprecipitation (ChIP)[13]. However, these experimental methods are usually time consuming and expensive. Therefore, the alternative computational methods are necessary for high-throughput identification of protein acetylation sites.

Currently, a large variety of computational methods have been proposed to predict acetylation sites based on the protein sequences. The majority of these methods are based on SVM classifier. For example, LysAcet [14], N-Ace [15], Ensemble-Pail [16], PLMLA [17], PSKAcePred [18], and BRABSB [19] which also combined bi-relative adapted binomial score Bayes. Additionally, the cluster based method, PredMod [20], combined experimental methods with sequences clustering analysis within histones. The logistic regression based method, LAceP [21], utilized logistic regression classifiers and integrated different biological features for lysine acetylation site prediction. The random forest based method, SSPKA [22], which firstly used functional features, was developed for species-specific lysine acetylation prediction. Particularly, Phosida [23,24] was firstly proposed as a well-known database containing various species-specific post-translational modification sites, and then developed into a high-accuracy species-specific acetylation site predictor.

Of note, there are several limitations in these methods. Firstly, for different species, the majority of existing methods just utilized an overall model to predict acetylation sites, without systematically discussing the differences of sequence and structure information among species. As we all known, different species may have different sequences or structures properties owing to different functional mechanism. Therefore, modelling based on different species may make improvements to acetylation prediction. Secondly, the methods in most of the papers only incorporated a small field of features, which would ignore some useful information, such as various predicted structural features. Finally, the sliding window strategy is often applied in feature extraction for obtaining the local information surrounding the lysine acetylation sites. Many studies only investigated a certain local sliding window in modeling. However, different sliding windows may have distinct prediction performances. Optimizing sliding window size is obviously helpful for selecting features and improving prediction performance.

**Fig 2:**
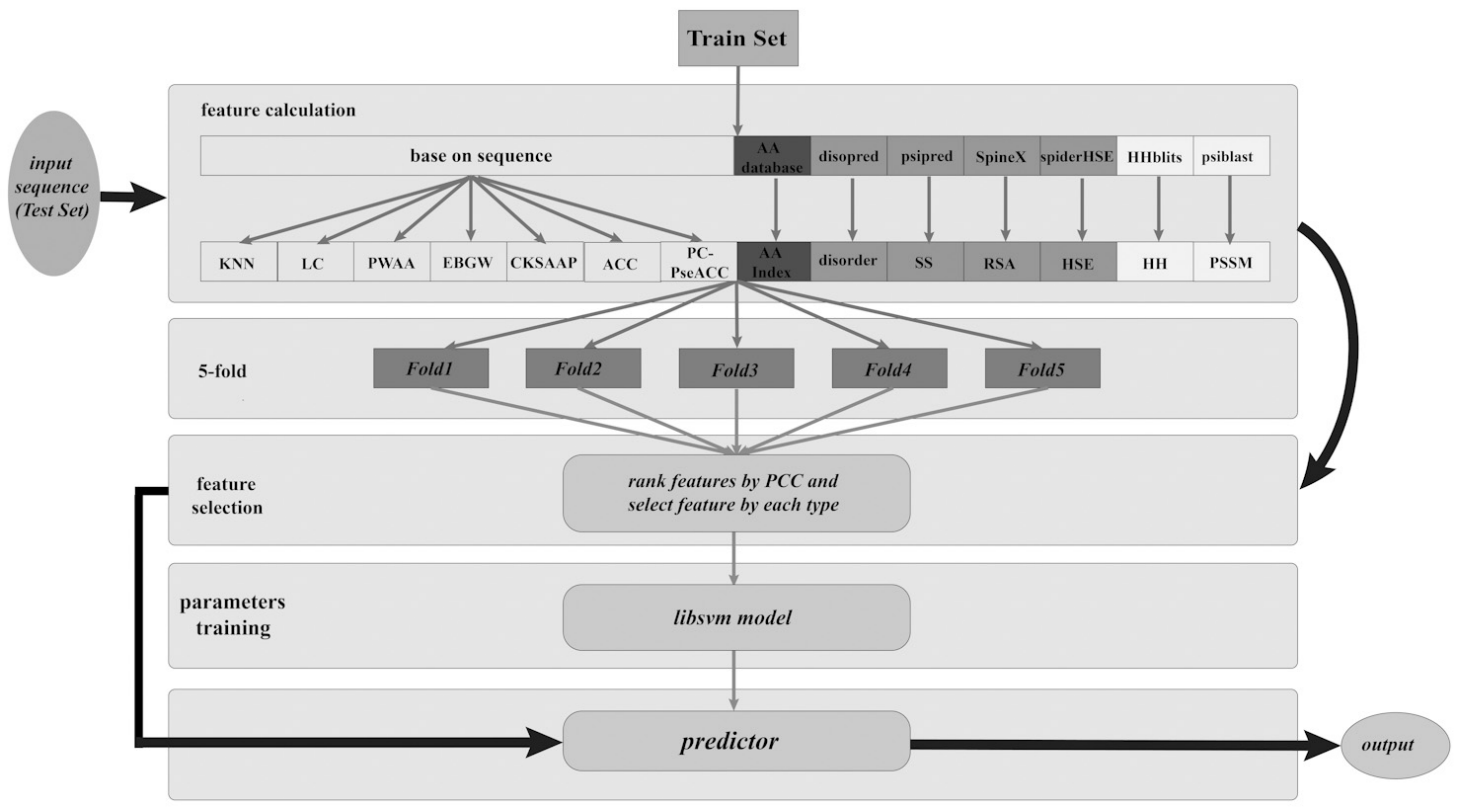
Flow chart of the KA-predictor approach, which includes feature calculation, feature selection and model training. Flowchart of training dataset is shown in grey arrow, and flowchart of test set is shown in black arrow. For training dataset, firstly, collecting 14 subtypes of features based on the training dataset by various tools, such as PSIBLAST and spider-HSE. Subsequently, ranking each type of features by the Pearson Correlation Coefficient (PCC) and conducting stepwise feature selection for each type. The 5-fold cross-validation was applied to feature selection by evaluating the performance on the training dataset. A support vector machine (SVM) classifier, LibSVM, was utilized to train parameters and build an accuracy prediction model. For independent test set or an input sequence for user, firstly conducting feature calculation and then selecting the same features as training dataset, finally utilizing the models trained on the training dataset to obtain the predicted output.

Aiming at these limitations, we developed a novel tool to predict species-specific lysine acetylation sites named as KA-predictor (lysine (K) Acetylation predictor) based on a support vector machine (SVM) classifier. In this study, we incorporated a large variety of features consisting of sequence-based features, physicochemical and biochemical properties features, predicted structural features and evolutionary information features. Particularly, the HSE features, which belonged to predicted structural features, were firstly introduced for lysine acetylation prediction and turned out to be fundamentally important in improving the predictive performance. Our predictor was designed for four species, i.e., *H*. *sapiens*, *M*. *musculus*, *E*. *coli*, and *S*. *typhimurium*. In addition, a sliding window strategy was applied in our study and the optimization of the sliding window sizes was used for selecting features and improving prediction performance. Furthermore, the prediction performances of our approach were compared with the latest methods in independent test set. The results indicated that our predictor was highly competitive for the majority of species when compared with other existing methods. Particularly, we firstly constructed a high-accurate structure dataset of *H*.*sapiens* to analyze the structural properties around lysine acetylation sites. A user-friendly local tool of KA-predictor can be freely downloaded at http://sourceforge.net/p/ka-predictor for the wider scientific community. A flowchart of the KA-predictor approach was given in **Fig 2**.

## Materials and Methods

### Datasets

In this study, we used the datasets from the study of SSPKA[22] which consisted of 1,936 non-redundant acetylation proteins with 3,956 acetylation sites of six species, i.e., *H*. *sapiens*, *M*. *musculus*, *E*. *coli*, *S*. *typhimurium*, *S*. *cerevisiae* and *R*. *norvegicus*, ranging from prokaryotes to eukaryotes. These datasets were extracted from multiple public resources including CPLA[25] (http://cpla.biocuckoo.org/), N-Ace[15] (http://N-Ace.mbc.NCTU.edu.tw/), Phosida[23] (http://www.phosida.com/), ASEB[26] (http://cmbi.bjmu.edu.cn/huac) and PhosphoSitePlus[27] (http://www.phosphosite.org). And these datasets had excluded the redundant sequences at the 30% identity level using CD-HIT[28] software. In order to consider the background proteins relative to acetylated proteins, SSPKA also provided a dataset of non-acetylated proteins (proteins that were not shown to be acetylated to date).

Here, we did not utilized the sequence data of non-acetylated proteins in SSPKA because the dataset contained some deleted or duplicated sequences and was of little reliability. Additionally, the data of *S*. *cerevisiae* and *R*. *norvegicus* was removed from our analysis because these two species had too few samples for training model (**Table 1**). Also, we discarded two protein sequences of A2ASS6 and Q8WZ42 in the training dataset since these two sequences are too long to run PSI-BLAST for calculating certain features such as PSSM. Finally, our dataset incorporated 1,870 of acetylation proteins with 3,790 acetylation sites of four species. In detail, the positive samples (i.e., acetylation sites) in the training dataset of our method were the same as the SSPKA method. To perform 5-fold cross-validation, the balanced negative samples (i.e., non-acetylation sites) were randomly extracted with the ratio of 1:1 of positive versus negative samples. (In fact, the amount of lysine acetylation sites is relatively small when compared with the amount of non-acetylated lysine residues in sequences.) For fair comparison, we used the identical positive and negative samples with SSPKA on independent test set. Since high similar data would lead to overestimate the accuracy of a method [29–32], we examined that the sequences in the training dataset were non-redundant with a threshold of 30% identity using the BlastClust tool. Furthermore, we examined that there were also non-redundancy between the sequences in training dataset and sequences in independent test set, so the independent test set was really independent from our training dataset. **Table 1** showed the statistics of training dataset and independent test set among four species.

**Table 1:** Statistics of acetylated proteins and sites information in training dataset and independent test set among four species, i.e. *H*. *sapiens*, *M*. *musculus*, *E*. *coli*, and *S*. *typhimurium*. (The non-acetylated sites were selected at the ratio of 1:1 compared to acetylated sites.) Additionally, the statistics data of *S*. *cerevisiae* and *R*. *norvegicus* was marked with asterisk (*) which means that we removed them from our analysis because these two species had too few samples for training model.

Species	Training dataset		Independent test set	
	Acetylated proteins	Acetylated sites	Acetylated proteins	Acetylated sites
*H*. *sapiens*	930	1885	190	477
*M*. *musculus*	341	744	84	188
*E*. *coli*	119	195	24	51
*S*. *typhimurium*	147	200	35	50
*S*. cerevisiae*	*35*	*69*	*9*	*17*
*R*. norvegicus*	*15*	*52*	*5*	*19*
Total	1537	3024	333	766

Particularly, we constructed a high-accurate structure dataset of *H*.*sapiens* from Protein Data Bank (PDB) [33–35] to analyze the structural properties around lysine acetylation sites. For *H*. *sapiens*, we collected 592 PDB structure records corresponding to sequences in our training dataset and independent test set. Because the incomplete or inaccurate structures were poor for structural properties analysis, we selected a subset of these PDB structures if the length of the PDB structure was longer than the 90% of the true length of corresponding sequence and resolution of the structure was less than 3Å. Finally, we obtained 118 PDB structures and then eliminated noises by manually aligning these structures to the corresponding sequences. Our high-accurate *H*.*sapiens* structure dataset included 308 lysine acetylation sites from 118 PDB structures. The reason for not constructing structure dataset for other species is that the experimentally determined crystal structures for other species were much fewer than *H*.*sapiens* and insufficient dataset would reduce the reliability of the analysis of structural properties. The datasets can be downloaded at http://sourceforge.net/p/ka-predictor.

### Features

In this study, we incorporated a large variety of features to predict acetylation sites. These features included four types: sequence-based features, physicochemical and biochemical properties features, predicted structural features and evolutionary information features. These can be further divided into 14 subtypes: LC, PWAA, EBGW, CKSAAP, KNN, ACC, PC-PseACC, AAindex, SS, RSA, Disorder, HSE, PSSM and HH (see below). Particularly, we firstly introduced the predicted half sphere exposure (HSE) as a type of features, which turns out to be fundamentally important in acetylation prediction. Additionally, we used a novel HMM-based tool, HHblits[36], to build protein multiple-sequence alignments (MSAs) for deriving position-specific scoring matrix and evolutionary conservation score, which is considered somewhat faster compared to PSI-BLAST[37]. A sliding window strategy was applied in feature extraction for obtaining the local information surrounding the lysine acetylation sites.

#### Sequence-based features

Location Coding (LC). For the sites located in the N-terminal, C-terminal or the middle of a sequence, we used 3-bit binary to encode this terminal information, i.e. N-terminal for 100, C-terminal for 001, middle for 010.

Position Weight Amino Acid Composition (PWAA). Position weight amino acid composition (PWAA) [17,38] is designed to avoid losing the sequence-order information of amino acid residues around certain sites. The position information of an amino acid in the sliding window can be calculated by the following formula:

$$C_{i}=\frac{1}{L(L+1)}\sum_{j=-L}^{L}x_{i,j}(j+\frac{\left|j\right|}{L})$$

where *L* denotes the number of upstream residues or downstream residues from the central site in the sliding window, *x*_*i*,*j*_ = 1 if *a*_*i*_ is the *j*-th residue in the sliding window, otherwise *x*_*i*,*j*_ = 0.

Encoding Based on Grouped Weight (EBGW). Encoding based on grouped weight (EBGW) [17,38,39] is an encoding scheme of the amino acid sequence based on the hydrophobicity and charged character of amino acid residues.

Firstly, 20 amino acid residues were divided into four different classes as follows: hydrophobic group *C1* = {*A*,*F*,*G*,*I*,*L*,*M*,*P*,*V*,*W*}, polar group *C2* = {*C*,*N*,*Q*,*S*,*T*,*Y*}, positively charged group *C3* = {*H*,*K*,*R*}, and negatively charged group *C4* = {*D*,*E*}. Then, we calculated three binary sequences of a certain sliding window:

$$H1(a_{j})=\{\begin{array}{c} 1\;if\;a_{j}\in C1\cup C2\\ 0\;if\;a_{j}\in C3\cup C4 \end{array}$$

$$H2(a_{j})=\{\begin{array}{c} 1\;if\;a_{j}\in C1\cup C3\\ 0\;if\;a_{j}\in C2\cup C4 \end{array}$$

$$H3(a_{j})=\{\begin{array}{c} 1\;if\;a_{j}\in C1\cup C4\\ 0\;if\;a_{j}\in C2\cup C3 \end{array}$$

where *a*_*j*_ represents the *j*-th residue in the sliding window sequence.

For each binary sequence, we could calculate *K* feature values based on *K* sub-sequences increasing in length as follows:

$$X(k)=\frac{sum(k)}{Int(N\cdot k/K)},k=1,2,\ldots ,K$$

Where the function *sum*(*k*) gives the number of 1 in the *k*-th sub-sequence, *Int*(*N*·*k*/*K*) denotes the length of the *k*-th sub-sequence, the *Int*() rounds a number to the nearest integer and *N* is the length of the sliding window sequence. Here, we made *K* = 5.

CKSAAP. The CKSAAP[40] encoding scheme means the composition of *k*-spaced residue pairs in the sliding window. In this study, we took *k* = 0. Therefore, there were 400 compositions of 0-spaced residue pairs, which could be calculated by

$${\left(\frac{N_{AA}}{N_{Total}},\frac{N_{AC}}{N_{Total}},\frac{N_{AC}}{N_{Total}},\ldots ,\frac{N_{YY}}{N_{Total}}\right)}_{400}$$

where the *N*_*Total*_ represents the total number of residue pairs in the sliding window, and the *N*_*XX*_ is the number of the residue pair XX in the sliding window.

K Nearest Neighbors (KNN) Score. To take advantage of the cluster information of local sequence fragments for predicting acetylation sites, we took a *K* nearest neighbors (KNN) score algorithm [18,41].

Firstly, we found the *K* nearest neighbors of a residue in both positive and negative datasets (training dataset was used in this paper). In detail, the distance between two local sequence fragments *S*_1_ and *S*_2_ defined as:

$$D(S_{1},S_{2})=1-\frac{\sum_{i=-n}^{n}sim(S_{1}(i),S_{2}(i))}{2L+1}$$

$$sim(a,b)=\frac{M(a,b)-\min(M)}{\max(M)-\min(M)}$$

where *a* and *b* are the two amino acid residues, *M* represents the substitution matrix of BLOSUM62[42], and the sliding window size is 2*L*+1.

After that, the corresponding KNN score was then extracted as follows: (i) Calculate the average distance from the sequence fragment *S* to the training dataset (contain the positive and negative datasets); (ii) Sort the neighbors by the distances and choose the *K* nearest neighbors; (iii) Calculate the percentage of positive neighbors in its *K* nearest neighbors as the KNN score.

Last, we chose five different *K* values, i.e., 1/2, 1/4, 1/8, 1/16 and 1/32 of the size of the training dataset.

Auto-cross covariance (ACC). Pse-in-One [43] is a recently constructed web server for users to generate all the possible pseudo components for DNA, RNA, and protein sequences, which combined different kinds of approaches [44–46]. In this paper, we used a combination of auto covariance and cross covariance measure based on the sliding window as features for lysine acetylation prediction.

Parallel correlation pseudo amino acid composition (PC-PseAAC). PC-PseAAC[47] is an approach incorporating the contiguous local sequence-order information and the global sequence-order information. Here, we calculated these features from Pse-in-One[43] as features for lysine acetylation prediction.

#### Physicochemical and biochemical properties features

AAindex. AAindex database[48] provides numerical indices that describe various physicochemical and biochemical properties of amino acids. For each index, we could express the physicochemical and biochemical information in the sliding window with 2L+1amino acid residues by the following formula:

$$A=\frac{1}{2L+1}\sum_{j=-L}^{L}p_{j}$$

where *p*_*j*_ is the index value of the *j*-th residue in the sliding window.

Additionally, Atchley et al.[49] summarized five highly representative indices, i.e., electrostatic charge, codon diversity, molecular volume, secondary structure and polarity, based on the AAindex database. We also used this information to encode each amino acid residue for determining the acetylation sites in this study.

#### Predicted structural features

Predicted Secondary Structure (SS). PSIPRED[50] is a neural-network-based secondary structure prediction tool, which shares a relatively high accuracy. Its outputs have three kinds of secondary structures: H (alpha-helix), E (beta-strand) and C (coil). In this paper, we used 3-bit binary to encode these three types, i.e. H for 100, E for 001, C for 010.

Predicted Solvent Accessibility (RSA). SPINE-X[51] is an accurate multistep neural-network method which can predict secondary structure, solvent accessibility and backbone torsion angles. In this paper, we used the values of RSA, psi and phi as features for lysine acetylation prediction.

Predicted Disorder Scores. DISOPRED2 [52,53] is proposed for recognizing natively disordered regions based on amino acid sequence. Based on the predicted values of this method, each residue can be divided into two types: disordered and ordered. In this paper, we used 1-bit binary to encode the feature, i.e. disorder for 1 and order for 0.

Predicted Half Sphere Exposure (HSE). Half sphere exposure (HSE) is firstly introduced by Hamelryck[54] to measure the solvent exposure of a protein, which plays a fundamentally important role in predicting discontinuous B-cell epitopes[5,55]. The measure means the number of C_α_ atoms in two half spheres around a residue's C_α_ atom. On the one hand, HSE can be classified as HSEA and HSEB based on whether only the information about the C_α_ atoms is available. On the other hand, it can be also divided into HSE-up (HSEU) and HSE-down (HSED) depending on the selected C_α_ atoms is in an up half-sphere (U) or a down half-sphere (D). SPIDER-HSE[56] is a consistent performance method designed for predicting both HSEA and HSEB of each residue in a certain protein.

Here, we firstly introduced each type of predicted HSE (i.e., HSEAU, HSEAD, HSEBU and HSEBD) information in a sliding window to predict lysine acetylation sites, which turn out to be fundamentally important in acetylation prediction.

Evolutionary information features. Position-Specific Scoring Matrix (PSSM) by PSI-BLAST. Position-specific scoring matrix (PSSM) can be calculated from multiple sequence alignment containing the evolutionary information of protein sequences. In this part, the PSSM for each sequence was generated by PSI-BLAST[37] based on NR database. The PSI-BLAST program can generate two types of position-specific scoring matrices, conservation scores and probabilities of occurrences. We used both of them to encode for predicting lysine acetylation sites.

Additionally, we calculated the evolutionary conservation scores which is defined as:

$$ECS(i)=-\sum_{j=1}^{20}p_{i,j}\log_{2}\left(p_{i,j}\right)$$

Where *p*_*i*,*j*_ represents the probability of amino acid *j* at position *i* of the sliding window (the sliding window size is 2*L*+1).

Position-Specific Scoring Matrix by HHblits (HH). HHblits[36] is an open-source, general-purpose tool that can build protein multiple-sequence alignments (MSAs) by profile hidden Markov models (HMMs), which is considered somewhat faster than PSI-BLAST. Here, we employed this novel tool to obtain the probabilities of occurrence, and then calculated the evolutionary conservation scores as mentioned above in prediction.

#### Support vector machine (SVM) classifier

Support vector machine (SVM) classifier was widely used in the statistics and bioinformatics [57–63], especially for functional site prediction such as acetylation, phosphorylation, ubiquitination and methylation [14–20,64–68]. SVM is a supervised learning model for binary classification by mapping the input samples to a higher dimensional space and searching a hyper-plane to distinguish the samples. In this paper, we applied the widely used SVM classifier, LibSVM (https://www.csie.ntu.edu.tw/~cjlin/libsvm/), to train parameters and build an accuracy prediction model. The version 3.14 of LibSVM was utilized. A radial basis function (RBF) was used as the type of kernel function, and two parameters, cost and gamma, were trained based on the parameter selection tool grid.py in LibSVM. To select features and evaluate the performance of the models, 5-fold cross-validation was performed. In statistical prediction, the independent dataset test, subsampling or K-fold crossover test and jackknife test are the three cross-validation methods often used to check a predictor for its accuracy [69]. However, among the three test methods, the jackknife test is deemed the least arbitrary that can always yield a unique result for a given benchmark dataset [70]. Accordingly, the jackknife test has been increasingly used and widely recognized by investigators to examine the quality of various predictors [71–75]. However, for saving computational time, the 5-fold cross-validation was used in the study.

### Feature selection method based on different type of features

As irrelevant or redundant features may lead to an adverse impact on prediction[76], we performed feature selection based on a large variety of features to remove redundant features and improve prediction performance. These features incorporated four types: sequence-based features, physicochemical and biochemical properties features, predicted structural features and evolutionary information features. These can be further divided into 14 subtypes: LC, PWAA, EBGW, CKSAAP, KNN, ACC, PC-PseACC, AAindex, SS, RSA, Disorder, HSE, PSSM and HH.

Firstly, we ranked each subtype of features by calculating the Pearson Correlation Coefficient (PCC) between each feature vector and the true classification index vector on training dataset. Pearson Correlation Coefficient (PCC) measures the linear correlation between two variables, giving a value between −1 and +1, where +1 represents total positive correlation, 0 represents no correlation, and −1 represents total negative correlation. It is widely used in the statistics and sciences. In this step, we obtained 14 PCC-ranked lists corresponding to 14 subtypes of features.

Then, for each subtype of features, we took a stepwise feature selection based on the support vector machine (SVM) classifier. At each round of stepwise feature selection, the next feature from the PCC-ranked list was added to the model if the accuracy of 5-fold cross-validation increased. After this step, we obtained the 14 optimal feature sets corresponding to 14 subtypes of features and then combined them to form the final selected features which were used to build model for predicting acetylation sites. The parameters of SVM classifier were trained based on the training dataset, using the grid selection tool in LibSVM.

This feature selection method based on different types of features, combining PCC ranking and stepwise feature selection, provides a practical approach for selecting a useful feature set.

### Performance Evaluation

In this paper, we utilized the 5-fold cross-validation on the training dataset to select features and build prediction models by SVM classifier. Then, we compared our predictor with other existing methods on the independent test set. Matthews correlation coefficient (*MCC*), accuracy (*ACC*), sensitivity (*SEN*), specificity (*SPE*), precision (*PRE*) and area under the receiver operating characteristic curve (*AUC*) were applied to evaluate performance of our acetylation predictor on training dataset and independent test set. Among them, *AUC* is calculated by the area under the receiver-operating characteristic (*ROC*) curve which is the major performance to estimate a classifier or method. Others are defined as:

$$MCC=\frac{TP\times TN-FP\times FN}{\sqrt{\left(TP+FP)(TP+FN)(TN+FP)(TN+FN\right)}}$$

$$ACC=\frac{TP+TN}{TP+TN+FP+FN}$$

SEN=TP/(TP+FN)

SPE=TN/(TN+FP)

PRE=TP/(TP+FP)

where *TP*, *TN*, *FP* and *FN* are defined as the numbers of true positives, true negatives, false positives and false negatives, respectively.

## Results

### Analysis of Compositional biases around acetylation sites

**Fig 3:**
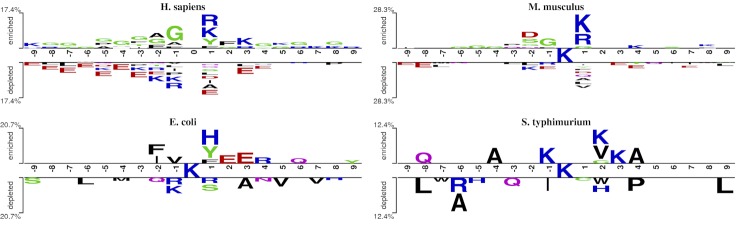
The compositional biases around the acetylation sites compared to the non-acetylation sites based on the two-sample logo[77]. Only amino acid residues significantly enriched or depleted (t-test for *p*-value < 0.05) around lysine acetylation sites are shown.

For different species, we adopted a web-based tool, Two Sample Logo[77], to present the compositional biases of sequences between acetylated and non-acetylated sites (**Fig 3**). Two Sample Logo is used to detect and visualize statistically significant differences in position-specific symbol composition between two sets of aligned samples of amino acids or nucleotides.

As we can see from **Fig 3**, each logo contains 19 residue fragments with 9 upstream and 9 downstream, based on the total datasets and there were some significant differences among different species. For example, the hydrophobic residues glycine (G) was enriched for *H*. *sapiens* and *M*. *musculus*; while the negatively charged residues, glutamic acid (E) and aspartic acid (D), were depleted for these two species. Interestingly, the lysine (K) was enriched at upstream fragment and depleted at downstream fragment for *H*. *sapiens* and *M*. *musculus*; while it was enriched in both upstream and downstream fragment for *S*. *typhimurium*. The positively charged residue arginine (R) was enriched at upstream fragment for *H*. *sapiens* and *M*. *musculus*, while it was depleted at downstream fragment for *H*. *sapiens*, *E*. *coli* and *S*. *typhimurium*.

Generally, the enriched residues and depleted residues had distinctly differences between species. These observations suggested that lysine acetylation sites among different species have distinct location-specific differences. Therefore, building a species-specific predictor was necessary and significant.

### Determination of the Sliding Window Sizes

Since different sliding windows may have distinct prediction performances, optimization of the sliding window sizes is required for selecting features and training models. On the one hand, if the sliding window was too long, a large amount of redundant information would be included. On the other hand, if the sliding window was too short, a lot of valuable information would lose. Thus, we took into account the window size varied from 11 to 19.

In this study, we used the predicted accuracy as index to evaluate the performance of the sliding windows with different sizes. Support vector machine (SVM) classifier and 5-fold cross-validation were carried out to build model and select feature based on each sliding window size.

**Fig 4:**
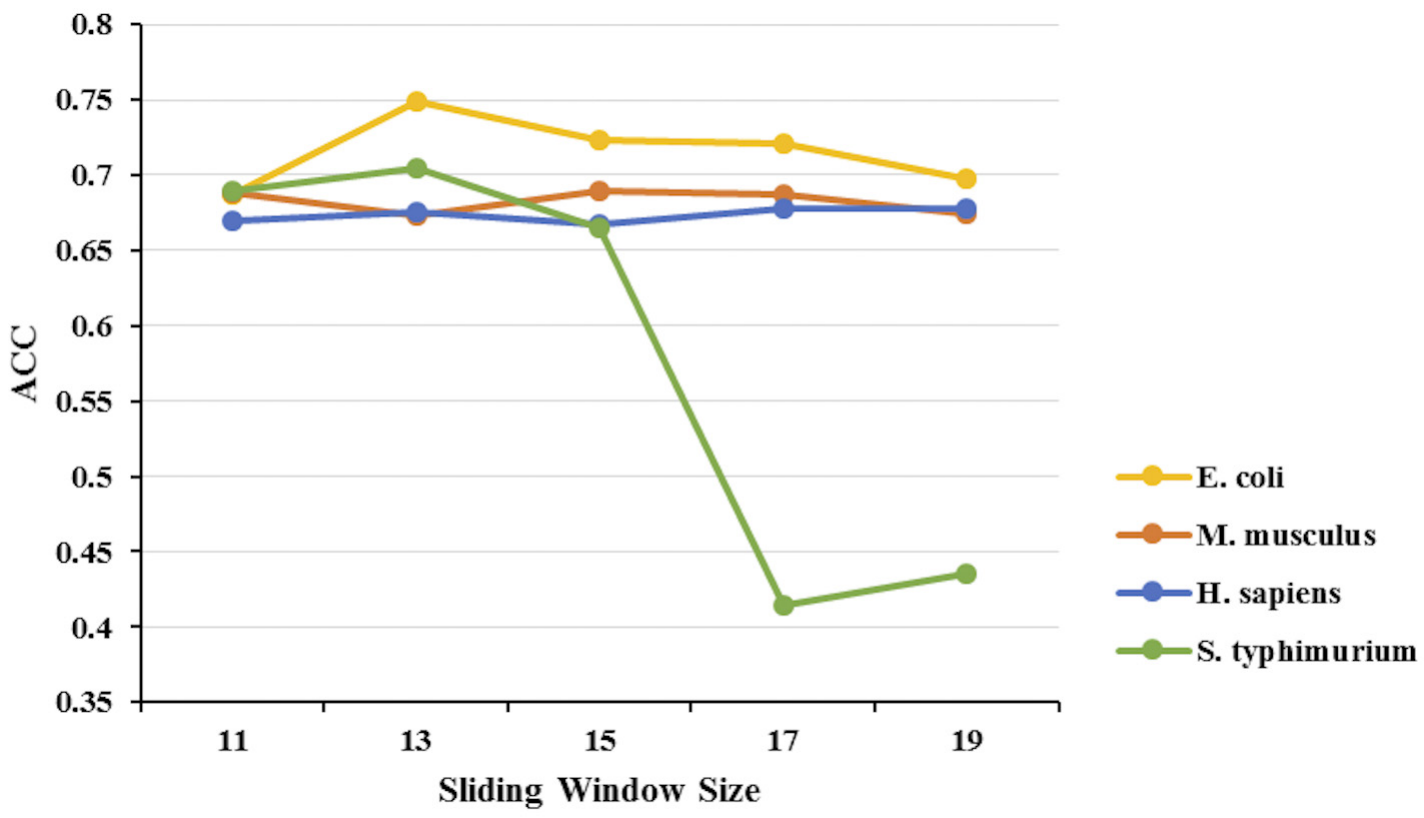
Predicted accuracy of 5-fold cross-validation based on the training dataset for sliding window size ranging from 11 to 19.

**Fig 4** showed the predicted accuracy of each model based on different window size. For *S*. *typhimurium*, the window size of 17 had the accuracy scores of 41.50%, while the accuracy score of the window size of 19 was 44.00%. The results showed these two window sizes were not stable. Therefore, we discarded the window sizes 17 and 19. The model with a window size of 13 had a relatively higher accuracy for most species than the window size of 11 and 15. Only for *M*. *musculus*, the predicted accuracy of window size of 13 was a little lower than the window size of 11 and 15. Compared with the window size of 11, the model trained using the window size of 13 improved 6.15%, 0.58% and 1.50% for *E*. *coli*, *H*. *sapiens* and *S*. *typhimurium*, respectively. And compared with the window size of 15, the window size of 13 improved 2.56%, 0.79% and 4.00% for *E*. *coli*, *H*. *sapiens* and *S*. *typhimurium*, respectively. According to these results, the optimal sliding window size was selected as 13 in our study.

### Predictive Capability of Different Types of Features

We integrated 14 subtypes of features and selected useful features from each type by combining PCC ranking and stepwise feature selection. To evaluate the predictive capabilities of different subtypes of features, we used the predicted accuracy as index. Here, we built 14 models corresponding to 14 subtypes of features using support vector machine (SVM) classifier. And then, we evaluated the performance of each model on training dataset and independent test set.

**Fig 5:**
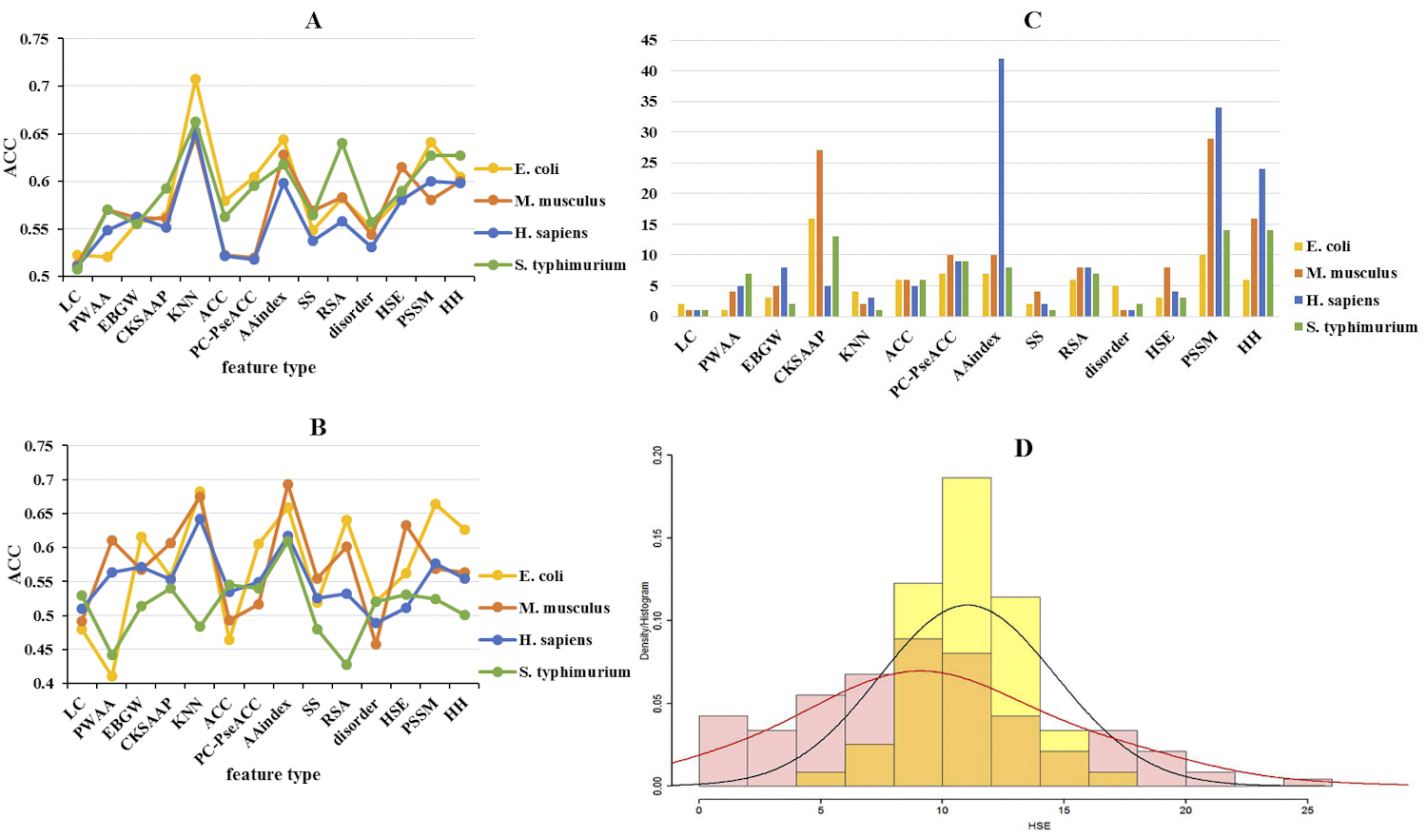
Predictive Capability of Different Types of Features on training dataset and independent test set. (A) Predicted accuracy of the models based on different types of features on training dataset for four species. (B) Predicted accuracy of the models based on different types of features on independent test set for four species. (C) The number of different types of features in the final selected featues set for four species. (D) Histograms and fitted density curves of HSEBU values between acetylation sites and non-acetylation sites on *H*. *sapiens* structure dataset. The histogram of acetylation sites is colored by pink and density curve is colored by red while the histogram of non-acetylation sites is colored by yellow and density curve is colored by black.

**Fig 5A** and **Fig 5B** showed the predicted accuracy of different models for four species based on 14 subtypes of features. For sequence-based features, the model trained with KNN features outperformed all of the others for all four species on training dataset; while the performances of the IC features were relatively poor. Additionally, the AAindex features had relatively higher accuracy scores for *H*. *sapiens*, *M*. *musculus* and *S*. *typhimurium* on training dataset. For predicted structural features, the accuracy score of the model trained by HSE features was relatively higher on training dataset, especially for *M*. *musculus*. In contrast, the performances of SS features and disorder features were relatively poor for *H*. *sapiens*. These results indicated that the contributive features on the training dataset were predominantly KNN, AAindex, HSE and PSSM. For independent test set, the accuracy score of AAindex features was the best for *M*. *musculus* and *S*. *typhimurium*, while the accuracy score of KNN features was the best for *E*. *coli* and *H*. *sapiens*. In contrast, the KNN features performed relatively poor for *S*. *typhimurium*. For predicted structural features, the HSE features had a relatively high accuracy scores with > 50% for all species; while the disorder features performed relatively poor. Interestingly, PSSM features had slightly higher performance than HH features for *H*. *sapiens*, while PSSM features were inferior to HH features for *M*. *musculus* on training dataset, suggesting that HH features, enjoying the advantage of faster calculation, also played a significant role in improving prediction performance as PSSM features.

As observed from **Fig 5C**, the number of selected optimal features differed, depending on different species. For example, *H*. *sapiens* had the largest set of optimal features with a total of 151 features, while *E*. *coli* had the smallest set with 78 features used to build model. The evolutionary information features (PSSM and HH features) took a relatively large proportion for all of the four species. Also, different species had distinct biases toward a certain type of features. For *H*. *sapiens*, the number of AAindex features was the largest. In contrast, the LC and disorder features were the smallest in number. Additionally, the number of CKSAAP was relatively large for *M*. *musculus* and *E*. *coli* while it was relatively small for *H*. *sapiens*. These results indicated that the contributive features for different species showed some significant differences.

More importantly, the predicted HSE features showed an outstanding performance on both training dataset and independent test set. The accuracy scores on independent test set were >50% for all of the species. For training dataset, the accuracy scores were also >50%. To examine the statistical significance of the HSE features between acetylation sites and non-acetylation sites, we further performed Wilcoxon signed-rank test based on training dataset (**Table 2**). The results showed that the differences of the HSE features between acetylation sites and non-acetylation sites were statistically significant for most species.

**Table 2:** Wilcoxon signed-rank test comparison of the statistical significance of the HSE between the acetylation sites and non-acetylation sites for all four species, i.e., *H*. *sapiens*, *M*. *musculus*, *E*. *coli* and *S*. *typhimurium*. The tests were based on the training dataset. (*p*-value < 0.05 by Wilcoxon signed-rank test)

Species	HSEAU	HSEAD	HSEBU	HSEBD
*H*. *sapiens*	0.0050	2.1756e-18	0.0159	0.3466
*M*. *musculus*	0.0074	2.3397e-09	0.0012	8.3233e-08
*E*. *coli*	0.2886	0.1067	0.3876	0.8038
*S*. *typhimurium*	0.0436	0.0060	0.0096	0.0262

Furthermore, we calculated real HSEU (HSE in the upper half sphere) values with a sphere radius of 13Å based on the structure dataset of *H*. *sapiens* to investigate the real differences of the HSE features between acetylation sites and non-acetylation sites. The average of HSEAU values for acetylation sites was 8.15, while for non-acetylation sites was 9.21. And the average of HSEBU values for acetylation sites (9.72) was also significantly less than non-acetylation sites (11.07) (*p*-value = 0.0027 by Wilcoxon signed-rank single side test). Some of the results were shown in **Fig 5D**. As mentioned before, acetylation is a process that an acetyl group (CH_3_CO-) from Ace-CoA replacing the hydrogen atom in the epsilon-amino group (-NH_3_^+^) of side chain of lysine residue, and the side chain of lysine residue is located at the upper half sphere which contains C_β_ atom (**Fig 1B**). Therefore, we investigated the HSEU values in this part. The lower HSEU value meant less neighbor residues around lysine residue in the upper half sphere, which may result in more opportunity for the transferring an acetyl group (CH_3_CO-) to the epsilon-amino group (-NH_3_^+^) of lysine residue, i.e., improving the ability of acetylation. Therefore, as shown in **Fig 5D**, the HSEU values for acetylation sites were significantly lower. This may be why predicted HSE can be a potential type of features for predicting acetylation site.

### Prediction Performance on Training Dataset

We evaluated the prediction performance of our models based on the final selected features. The 5-fold cross-validation was used to evaluate the performance on the training dataset. The predicted performances were presented in **Table 3**. SVM models for all four species displayed relatively good performance with AUC scores ranging from 0.723 to 0.787. Among these species, the performances of the models for *E*. *coli* were the best with AUC scores of 0. 787. And the AUC score of *H*. *sapiens* was 0.737, while the performance of *M*. *musculus* was a little poor, with the AUC scores of 0.723. *M*. *musculus* had the poorest MCC, ACC, SEN, SPE and PRE scores of 0.342, 0.671, 0.685, 0.657 and 0.667 respectively. These results indicated that our predictor provided a good predictive ability on the training dataset.

**Table 3:** The prediction performance of KA-predictor based on 5-fold cross-validation on training dataset.

Species	MCC	ACC	SEN	SPE	PRE	AUC
*H*. *sapiens*	0.351	0.676	0.679	0.673	0.675	0.737
*M*. *musculus*	0.342	0.671	0.685	0.657	0.667	0.723
*E*. *coli*	0.416	0.708	0.687	0.728	0.717	0.787
*S*. *typhimurium*	0.386	0.693	0.720	0.665	0.682	0.756

### Independent test set and Comparison with Existing Methods

To further evaluate the performance of our predictor, we made comparison with the other existing methods on the independent test set. Here we put our independent test sets into 7 previously developed methods: LysAcet[14], ensemblePail[16], Phosida[23,24], PLMLA[17], PSKAcePred[18], BRABSB[19] and SSPKA[22]. As we utilized the same independent test set as SSPKA, the performance of other exiting methods were from SSPKA[22]. The comparison results of our predictor with other existing methods based on independent test set were shown in **Table 4**. As we can see, our predictor outperformed the majority of other methods for different species, such as *M*. *musculus* and *E*. *coli*. Particularly, our predictor achieved AUC score of 0.713 for *M*. *musculus* and 0.734 for *E*. *coli*, which was significantly higher than the others.

**Table 4:** Performance comparison of our predictor with other existing methods on independent test set. As we utilized the same independent test set as SSPKA, the performance of other exiting methods are from SSPKA[22].

Species	Methods	MCC	ACC	SEN	SPE	PRE	AUC
*H*. *sapiens*	PLMLA	0.296	0.648	0.633	0.663	0.667	0.689
	Phosida	0.136	0.568	0.553	0.583	0.585	0.597
	LysAcet	0.120	0.558	0.503	0.616	0.583	0.552
	ensemblePail	0.076	0.535	0.457	0.618	0.560	0.534
	PSKAcePred	0.111	0.556	0.553	0.558	0.571	0.556
	BRABSB	0.275	0.637	0.612	0.663	0.659	0.645
	SSPKA	0.214	0.600	0.482	0.725	0.652	0.606
	Our Predictor	0.257	0.629	0.696	0.558	0.626	0.657
*M*. *musculus*	PLMLA	0.182	0.590	0.521	0.659	0.609	0.604
	Phosida	0.035	0.517	0.516	0.519	0.522	0.525
	LysAcet	0.137	0.568	0.590	0.546	0.569	0.590
	ensemblePail	0.104	0.550	0.431	0.670	0.570	0.555
	PSKAcePred	0.282	0.635	0.511	0.762	0.686	0.652
	BRABSB	0.172	0.584	0.511	0.659	0.604	0.592
	SSPKA	0.222	0.611	0.638	0.584	0.609	0.661
	Our Predictor	0.314	0.657	0.648	0.665	0.663	0.713
*E*. *coli*	PLMLA	0.255	0.627	0.608	0.647	0.633	0.675
	Phosida	0.258	0.627	0.706	0.549	0.610	0.662
	LysAcet	0.045	0.520	0.275	0.765	0.538	0.440
	ensemblePail	-0.064	0.471	0.275	0.667	0.452	0.452
	PSKAcePred	0.020	0.510	0.412	0.608	0.512	0.492
	BRABSB	0.118	0.559	0.510	0.608	0.565	0.582
	SSPKA	0.321	0.657	0.549	0.765	0.700	0.687
	Our Predictor	0.375	0.686	0.745	0.627	0.667	0.734
*S*. *typhimurium*	PLMLA	0.101	0.550	0.600	0.500	0.545	0.520
	Phosida	0.000	0.500	0.560	0.440	0.500	0.442
	LysAcet	0.100	0.550	0.560	0.540	0.549	0.514
	ensemblePail	0.000	0.500	0.280	0.720	0.500	0.491
	PSKAcePred	0.120	0.560	0.560	0.560	0.560	0.504
	BRABSB	0.042	0.520	0.360	0.680	0.529	0.495
	SSPKA	0.222	0.610	0.540	0.680	0.628	0.581
	Our Predictor	0.040	0.520	0.560	0.480	0.519	0.542

**Fig 6:**
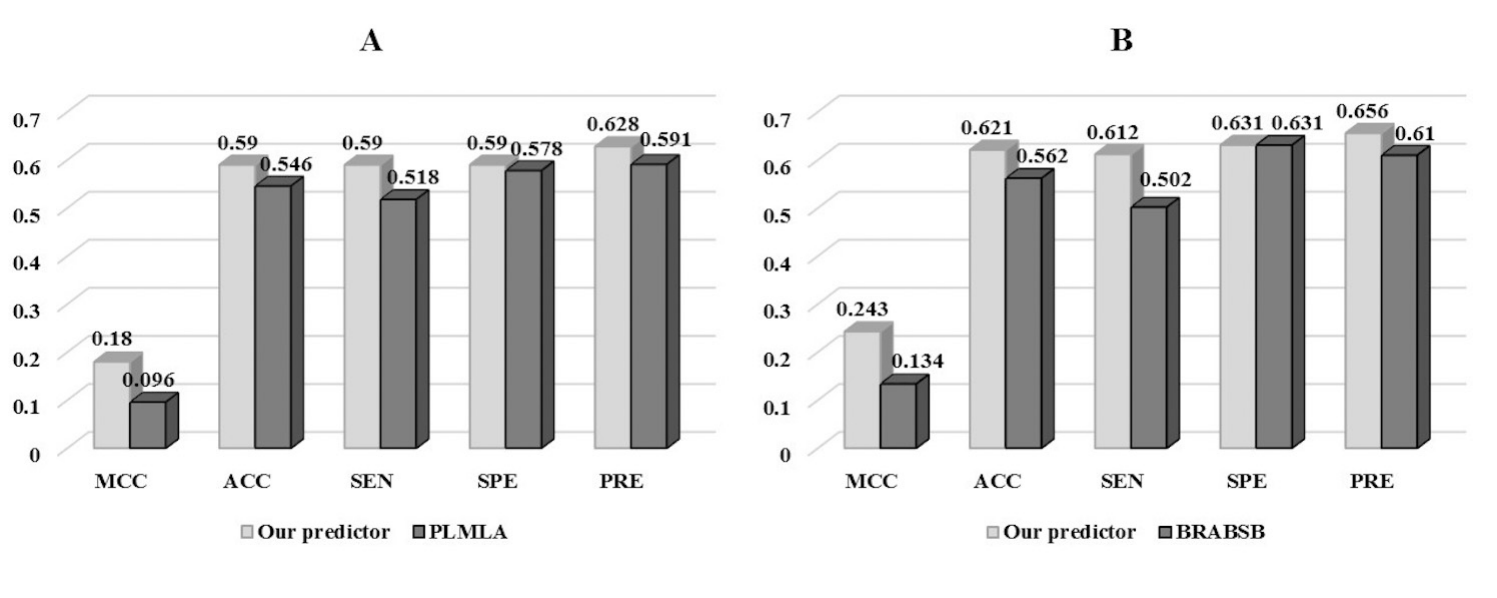
Further Comparison of our predictor with PLMLA and BRABSB for *H*.*sapiens*. (A) Comparison of our predictor with PLMLA. (B) Comparison of our predictor with BRABSB.

However, our method performed slightly lower AUC score than PLMLA and lower ACC score than BRABSB on independent test set. The reason was that the independent test set used in this paper was not blind to the training dataset of these two methods. Also, in order to remove interference made by homologous sequences and avoid overestimation, different methods selected different cutoff from 25% to 40%. For example, our independent test set for *H*. *sapiens* had 190 acetylation proteins, however, 107 sequences among these had the identity of >30% with the training set of BRABSB and 116 for PLMLA. For fair comparison, we used the rest sequences (83 for BRABSB and 74 for PLMLA) to compare the prediction performance with PLMLA and BRABSB, respectively. As shown in **Fig 6,**, our predictor apparently outperformed these two methods. When compared to BRABSB, our predictor achieved a MCC score of 0.243 and an ACC score of 0.621 while 0.134 and 0.562 for BRABSB, respectively. For PLMLA, the MCC score of our predictor was 0.180 when compared to 0.096 for PLMLA, and the ACC score of our predictor was 0.590 when compared to 0.546 for PLMLA.

In addition, the results of *S*. *typhimurium* were a little poor when compared with some methods. As mentioned above, different methods selected different samples as training dataset and independent test set. The training set of some existing methods may contain the information of our independent test set which results in the improvement of the performance for some methods.

These results have clearly indicated that our predictor was highly competitive when compared with other methods in predicting the acetylation sites for the majority of species.

### Comparison of different feature selection methods

Max-Relevance-Max-Distance (MRMD) [78] is a feature ranking method, which includes two main part of the decision: measuring the relevance between features in a subset by Pearson’s correlation coefficient (PCC), and calculating the redundancy among features in a subset through Euclidean distance, Cosine distance and Tanimoto. Minimum Redundancy Maximum Relevance (mRMR) was proposed for processing microarray data and then applied into other field [79,80]. The approach selects the features having minimal redundancy which means a new selected feature should have least redundancy in the remaining of features, as well as the maximal relevance which means selected feature should have the strongest relevance to the target type. The mutual information (MI), well known in probability theory and information theory, is a measure of the mutual dependence between the two variables.

As already described in the above, we ranked different type of features by calculating the Pearson Correlation Coefficient (PCC) between each feature and the true classification on training dataset. In order to understand the influence of different feature selection methods for prediction, we compared the performances of models built based on different feature selection methods on independent test set (**Table 5**). The PCC method performed excellently on most of the species, especially for *H*. *sapiens* and *M*. *musculus*. The MRMD had the best MCC score of 0.394 for *E*. *coli* and the best AUC score of 0.561 for *S*. *typhimurium*, while it performed worst for *H*. *sapiens*. The models based on the mRMR method performed worst for *S*. *typhimurium*, and MI method had relatively poor performance for *E*. *coli*. These results indicated that ranking features based on PCC can be helpful to selecting essential features for lysine acetylation prediction and improving the accuracy of predicting lysine acetylation sites.

**Table 5:** Performance comparison of different feature selection methods on independent test set.

Species	Selection Methods	MCC	ACC	SEN	SPE	PRE	AUC
*H*. *sapiens*	MRMD	0.180	0.591	0.667	0.511	0.592	0.625
	mRMR	0.196	0.599	0.652	0.542	0.603	0.632
	MI	0.230	0.616	0.690	0.538	0.614	0.653
	PCC	0.257	0.629	0.696	0.558	0.626	0.657
*M*. *musculus*	MRMD	0.255	0.627	0.670	0.584	0.621	0.686
	mRMR	0.303	0.651	0.660	0.643	0.653	0.705
	MI	0.206	0.603	0.601	0.605	0.608	0.683
	PCC	0.314	0.657	0.648	0.665	0.663	0.713
*E*. *coli*	MRMD	0.394	0.696	0.745	0.647	0.679	0.698
	mRMR	0.257	0.627	0.686	0.569	0.614	0.689
	MI	0.137	0.569	0.608	0.529	0.564	0.598
	PCC	0.375	0.686	0.745	0.627	0.667	0.734
*S*. *typhimurium*	MRMD	0.062	0.530	0.640	0.420	0.525	0.561
	mRMR	-0.201	0.490	0.620	0.360	0.492	0.503
	MI	0.081	0.540	0.600	0.480	0.536	0.508
	PCC	0.040	0.520	0.560	0.480	0.519	0.542

### Comparison of different classifiers

In this paper, we used the LibSVM classifier to predicting lysine acetylation sites. However, ensemble classifier was generally considered to outperform a simple classifier. So I compared the performance with a latest ensemble classifier, libD3C[81] employing two types of selective ensemble techniques, which are a combination of the ensemble pruning based on k-means clustering and dynamic selection and circulating combination. We applied the LibD3C to select features and build prediction model by the same methods as LibSVM. The comparison results of the LibSVM and LibD3C classifier based on independent test set were shown in **Table 6**. The performance of these two classifiers is pretty close. And LibSVM performed relatively better than LibD3C in H. *sapiens*, E. *coli* and S. *typhimurium*. For *M*. *musculus*, the LibD3C classifier shared a comparable performance with LibSVM.

**Table 6:** Performance comparison of LibSVM and LibD3C on independent test set.

Species	Classifiers	MCC	ACC	SEN	SPE	PRE
*H*. *sapiens*	LibD3C	0.165	0.584	0.652	0.511	0.587
	LibSVM	0.257	0.629	0.696	0.558	0.626
*M*. *musculus*	LibD3C	0.314	0.657	0.691	0.622	0.650
	LibSVM	0.314	0.657	0.648	0.665	0.663
*E*. *coli*	LibD3C	0.281	0.637	0.745	0.529	0.613
	LibSVM	0.375	0.686	0.745	0.627	0.667
*S*. *typhimurium*	LibD3C	0	0.500	0.560	0.440	0.500
	LibSVM	0.040	0.520	0.560	0.480	0.519

## Conclusion and Discussion

In this study, we developed a novel predictor, KA-predictor, which has significantly improved the prediction performance of species-specific lysine acetylation sites across four different species, i.e., *H*. *sapiens*, *M*. *musculus*, *S*. *typhimurium* and *E*. *coli*, by combining a variety of features. We incorporated different types of features and employed an efficient feature selection on each type to form the final optimal feature set for model learning. We evaluated the prediction performance of our models based on the final selected features. The training dataset showed a relatively good performance based on the 5-fold cross-validation. Additionally, the results on the independent test set indicated that KA-predictor was able to perform competitively, compared with existing tools. Feature contribution analysis indicated that HSE features, firstly introduced for lysine acetylation prediction, significantly improve the predictive performance. In addition, we compared different kinds of feature selection methods and different classifiers with ours. The results indicated that ranking features based on PCC and utilizing LibSVM classifier can be helpful to improving the accuracy of predicting lysine acetylation sites. Particularly, we constructed a high-accurate structure dataset of *H*.*sapiens* from PDB to analyze the structural properties around lysine acetylation sites. Moreover, a user-friendly tool was freely available at http://sourceforge.net/p/ka-predictor.

Although our predictor can perform accurately according to the above results, some issues must still be addressed in future work. Firstly, the number of negative samples (i.e. non-acetylation sites) was much larger than that of positive samples (i.e. acetylation sites) resulting in the problem of imbalanced learning. We randomly sampled a subset from the negative samples with a ratio of 1:1 of positive versus negative samples to form a relatively balanced training dataset. However, the information loss brought by random under-sampling will weaken the prediction performance. Ensemble learning method to reduce the impact of under-sampling is crucial for improving the prediction performance. Secondly, we used datasets from SSPKA[22] proposed about 2 years ago. There may have some new data in different databases. Also, the independent test set used in this paper was really blind to the training dataset, but may not be independent to other existing predictors. Hence, making full use of new data and constructing a test set that is truly independent to each predictor are important. Finally, a lot of other amino acid residues can be also acetylated such as, Alanine (A), Glycine (G), Methionine (M), Serine (S) and Threonine (T). In the future work, we can take into account the prediction of acetylation of these amino acids.
